# Generation of Granule Cell Dendritic Morphologies by Estimating the Spatial Heterogeneity of Dendritic Branching

**DOI:** 10.3389/fncom.2020.00023

**Published:** 2020-04-09

**Authors:** Zane Z. Chou, Gene J. Yu, Theodore W. Berger

**Affiliations:** Department of Biomedical Engineering, University of Southern California, Los Angeles, CA, United States

**Keywords:** dendrite, morphology, computational modeling, granule cell, point process

## Abstract

Biological realism of dendritic morphologies is important for simulating electrical stimulation of brain tissue. By adding point process modeling and conditional sampling to existing generation strategies, we provide a novel means of reproducing the nuanced branching behavior that occurs in different layers of granule cell dendritic morphologies. In this study, a heterogeneous Poisson point process was used to simulate branching events. Conditional distributions were then used to select branch angles depending on the orthogonal distance to the somatic plane. The proposed method was compared to an existing generation tool and a control version of the proposed method that used a homogeneous Poisson point process. Morphologies were generated with each method and then compared to a set of digitally reconstructed neurons. The introduction of a conditionally dependent branching rate resulted in the generation of morphologies that more accurately reproduced the emergent properties of dendritic material per layer, Sholl intersections, and proximal passive current flow. Conditional dependence was critically important for the generation of realistic granule cell dendritic morphologies.

## Introduction

The electrophysiological properties and spiking behavior of neurons are significantly affected by their dendritic morphologies (Mainen and Sejnowski, 1996; van Elburg and van Ooyen, 2010; Ferrante et al., 2013). Although the functional consequence of dendritic morphology at the individual cell level has been well-studied (Cuntz et al., 2013), the functional consequence of morphologies in a large network is not well-understood. Such large network studies require the use of many morphologies to account for the naturally occurring variations that exist among the population of each neuron type (van Pelt and Uylings, 2005). Numerous neural reconstructions exist that can be used for this purpose; digital tracings of prepared tissue from various neuroanatomical studies have been collected and made publicly available on online databases (Ascoli et al., 2007). However, the amount of reconstructions currently available is insufficient to specify the full numbers of neurons in a neural region and the particular morphologies that are included in these studies may not fully represent the systematic changes in morphology that can exist along an anatomical gradient within a neural region. To address these limitations, the field has made efforts to create algorithms that generate additional dendritic morphologies either by simulating the mechanisms through which dendrites grow or by measuring the distributions of key generative parameters from existing neural reconstructions and iteratively sampling from the distributions to create new morphologies (Torben-Nielsen and Cuntz, 2014).

Widely used generation tools such as L-Neuron (Ascoli and Krichmar, 2000) are capable of implementing various sampling algorithms, such as the Burke (Burke et al., 1992) or Hillman (Hillman, 1979) algorithms, to create morphologies with morphometrics attuned to the desired neuronal type. Important generative parameters—compartment diameter taper, daughter ratios, and others—are first measured from real morphologies using analytical tools (Scorcioni et al., 2008; Peng et al., 2015). From these measurements, histograms are generated and then fitted to probability distributions that describe statistics such as the mean and variance. Afterwards, dendritic morphologies are generated according to a flowchart decision tree. At necessary decision points, values are independently drawn from extracted distributions to construct the subsequent section of the dendritic tree. This process is repeated until a termination criterion is satisfied for all branches, usually based on whether the diameter of the branch falls below a certain threshold.

Though the existing algorithms have been successful in recreating many of the morphological features of specific neuron types, they can also generate unrealistic morphologies that need to be removed through a screening process (Schneider et al., 2012). This suggests that the rules underlying stochastic generation algorithms can result in combinations of parameter values that are not observed in the experimental datasets. A potential contributing factor is the fact that these stochastic sampling algorithms make a critical assumption that parameters are independent and memoryless, or in other words, a parameter does not depend on other factors or parameters and does not depend on previous realizations of the parameter.

There have been efforts to address this dilemma and add some context sensitivity to generation algorithms (Lindsay et al., 2007; Koene et al., 2009; Cuntz et al., 2010; Schneider et al., 2014; Torben-Nielsen and De Schutter, 2014; Beining et al., 2017; Kanari et al., 2018). Some of these, such as NETMORPH (Koene et al., 2009), incorporate dependence between variables by using trophic factors to influence branching and branch angles. The trophic factors cause branches to grow away from the soma and avoid other nearby branches and branches of neighboring neurons (Nowakowski et al., 1992; Samsonovich and Ascoli, 2003). However, this approach is computationally intensive and requires all neurons to be generated simultaneously within the desired three-dimensional space. It also requires heavy parameter optimization for the mechanisms of branching and elongation based on the environmental factors (Segev and London, 2000; Scott and Luo, 2001; van Pelt and Schierwagen, 2004; Jan and Jan, 2010).

Non-parametric approaches offer another promising solution to capture the dynamics of dendritic branching behavior while remaining agnostic to the exact mechanisms by which it occurs. Previous work along these lines by Torben-Nielson and colleagues used a kernel-based algorithm to generate dendritic morphologies (Torben-Nielsen et al., 2008). However, the authors further describe the need for a “truly conditional sampling procedure,” which would explicitly link the selection of parameter values to other parameter values and to previously selected values.

In this paper, we introduce a method for determining dendritic branching using a heterogeneous point process. A point process filter is used to determine the relations between branching rate, spatial location, and the history of branching, and the point process is incorporated into a modified Hillman framework to generate a hybrid generation approach that can account for dependencies between variables. We have named this method SHAPED: Spatially Heterogeneous Assignment of Parameters' Estimated Distributions and have applied the algorithm to generate realistic granule cell morphologies for the rat dentate gyrus. We demonstrate that by incorporating the heterogeneous nature of branching and branch orientation in the models, the resulting morphologies more accurately reproduce the emergent and functional properties observed in real morphologies compared to morphologies generated by algorithms that assume independence.

## Materials and Methods

### Dendritic Branching as a Heterogeneous Point Process

In granule cells, higher degrees of branching are observed in the most proximal third of the molecular layer, which has been attributed to a combination of the timing of the arrival of association and commissural afferents to the dentate gyrus and the maturity of the granule cell dendrites (Gottlieb and Cowan, 1973). Similar theories have been used to explain the higher percentage of total dendritic material residing in the middle and outer molecular third of granule cells (Rihn and Claiborne, 1990).

Ideally, the generation approach should capture the differences between the inner, middle, and outer thirds of the molecular layer, which are caused by the different quality and timing of inputs. The simplest method would be to separately measure the generative parameters for the dendrites in each layer. However, categorizing dendrites into layers a priori may not capture the appropriate changes in morphometric parameters and would be unable to reveal any variation that occurs within a layer. Discretizing the morphology using a bin size would introduce an additional variable that must be optimized with its own tradeoffs. For example, using smaller bins would provide more resolution but reduce the statistical power in each bin.

**Figure 1:**
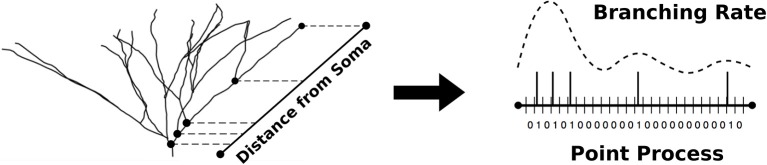
The non-parametric algorithm models dendritic branching as a point process with discretized pathlength. The optimal estimate of the branching rate is obtained and used to simulate point processes used in the generation of new morphologies.

To circumvent this issue, we instead modeled the branching rate variation as a heterogeneous Poisson process in which the Poisson process describes dendritic bifurcation events (Figure 1). To perform the estimation, we used a point process filter, a discrete analog of the Kalman filter that has been successfully implemented in neural decoding schemes to provide estimates of neural firing rates (Shanechi et al., 2012), among other applications. Here, we have applied the point process filter to the dendritic branching problem, in which observations of bifurcation events can be used to estimate the hidden state that dictates the frequency of those bifurcations, i.e., a branching rate.

**Figure 2:**
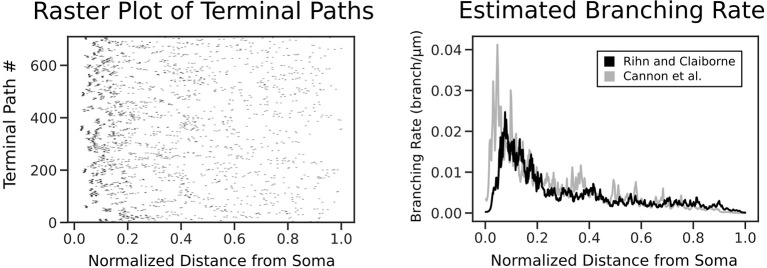
Each terminal path of the real morphologies is converted into a point process along a normalized axis of pathlength from the soma. The point process smoother provides the branching rate estimate shown on the right. The estimated branching rates are shown for two datasets: Rihn and Claiborne (1990) (black) and Cannon et al. (1998) (gray), with both demonstrating a higher branching rate at distances closer to the soma.

To formulate the morphology as a spatial point process, we identified every possible terminal path in a given morphology, from the soma to a dendritic tip. Each of these terminal paths were divided into discrete intervals of size Δ, where Δ was selected to be small enough such that there was never more than one bifurcation in the same interval. A point process was then constructed based on the occurrence of a bifurcation event within each interval, which was denoted with a 1 or 0 correspondingly. To assist in defining the extent of the terminal path, the dendritic tips were also treated as a bifurcation event and each point process, therefore, was assigned a 1 as its last value. Once all point processes had been prepared, we combined the point process filter and smoother with an expectation-maximization algorithm in order to simultaneously solve for the optimal branching rate estimate (Figure 2) and the necessary model parameters, as described by Smith and Brown (2003).

The state-space model used is an analog for a simplistic model of neuronal firing rate. The state model for the hidden variable x_k_, which is defined as the log of the branching rate, λ_k_, is a first order autoregressive Gaussian model with the state noise variance σ^2^. The observation model is a discretized point process n_k_ that describes the number of bifurcation events that have occurred in the interval (kΔ, kΔ + Δ), in which R is the total number of point processes in the data set and N is the total number of bifurcations in the point process.

$$\begin{array}{l} x_{k}=\rho x_{k-1}+\varepsilon_{k}\\ \varepsilon_{k}=N\left(0,\;\sigma_{\varepsilon }^{2}\right)\\ \lambda_{k}=exp\left(\mu +\beta x_{k}\right) \end{array}$$

The point process filter algorithm presented in Mendel (1995) and Brown et al. (1998) was used to obtain an estimate of the posterior density of the branching rate. This was done via a one-step prediction and recursive non-linear filtering to obtain the best estimate of x_k|k_.

$$\begin{array}{l} x_{k|k-1}=\rho x_{k-1|k-1}\\ \sigma_{k|k-1}^{2}=\rho^{2}\sigma_{k-1|k-1}^{2}\;+\;\sigma_{\varepsilon }^{2}\\ x_{k|k}=x_{k|k-1}+\sigma_{k|k-1}^{2}\sum_{r=1}^{R}\beta \left[dN^{r}\left(k\Delta \right)-exp\left(\mu_{c}+\beta x_{k|k}\right)\Delta \right]\\ \sigma_{k|k}^{2}=-{\left[-{\left(\sigma_{k|k-1}^{2}\right)}^{-1}-\sum_{r=1}^{R}\beta^{2}exp\left(\mu +\beta x_{k|k}\right)\Delta \right]}^{-1} \end{array}$$

In this application, the estimation can benefit from the inclusion of the complete data, since the growth of dendrites is not purely dependent on prior events but can change during later remodeling. A fixed interval smoother takes advantage of this extra information, and was used to obtain the smoothed state prediction, x_k|K_.

$$\begin{array}{l} x_{k|K}=x_{k|k}+A_{k}\left(x_{k+1|K}-x_{k+1|k}\right)\\ A_{k}=\rho \sigma_{k|k}^{2}{\left(\sigma_{k+1|k}^{2}\right)}^{-1}\\ \sigma_{k|K}^{2}=\sigma_{k|k}^{2}+A_{k}^{2}\left(\sigma_{k+1|K}^{2}-\sigma_{k+1|k}^{2}\right) \end{array}$$

Estimation of the branching rate and optimization of the parameters ρ, μ, β, and σ^2^ was performed recursively using the expectation-maximization algorithm (Smith and Brown, 2003).

### Dendritic Termination at the Hippocampal Boundaries

Another problem of traditional algorithms for dendritic morphology generation is that they commonly determine where a branch terminates by evaluating whether the branch diameter is smaller than a randomly drawn diameter threshold (Hillman, 1979). This practice suffers in cases with either extremely high or low variance in branch diameter and tapering measurements. Issues with low variance in some older studies can be compounded by the limited optical resolution available for some morphology data sets, which can lead to little or no taper being reported for dendrites because the differences were too small to be captured. In these circumstances, diameter is a poor indicator for branch termination.

As an alternative criterion for termination, we propose establishing a terminal boundary that is defined by the anatomy of the neural region, in our case the hippocampal fissure (for suprapyramidal granule cells) or the pial surface (for infrapyramidal granule cells). In the hippocampus, neuronal dendrites have been observed to proceed from the cell body layer to these boundaries, where the vast majority of all dendritic processes are observed to terminate. This means that the thickness of the hippocampus at a cell's location affects how long the dendritic arbor extends. This pattern of growth has been well-observed in many hippocampal subregions, including both CA3 (Gottlieb and Cowan, 1973) and dentate gyrus neurons (Gallitano et al., 2016), and accounts for differences in total dendritic length in the suprapyramidal blade and infrapyramidal blade due to the natural differences in hippocampal thickness in those regions.

To implement this new termination criterion, we first measured the distribution of all terminal pathlengths in the 43 morphological reconstructions of granule cells (Rihn and Claiborne, 1990). At the beginning of the generation step, a random value was selected from this distribution to determine the approximate location of the outer molecular layer with respect to the somatic location. This represents the expected terminal pathlength of the generated morphology. In a full simulation environment, the random selection can then be informed by the intended location of the generated neuron within the entire dentate structure in order to create morphologies in which size is dependent on realistic environmental constraints.

**Figure 3:**
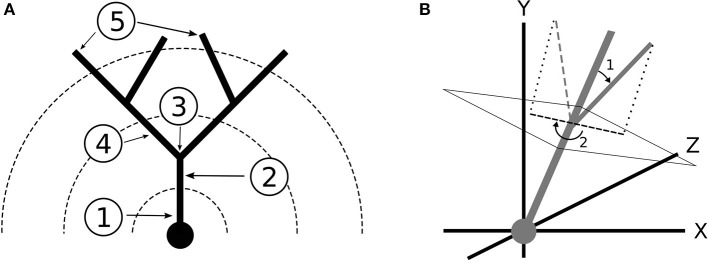
**(A)** Terminology for dendritic branching: (1) Soma (2) Stem (3) Bifurcation (4) Branch (5) Termination. The dotted lines represent Sholl radii. **(B)** Coordinate system and branch angles defined. The dendrites grow toward the positive y direction. (1) Bifurcation Amplitude (2) Bifurcation Azimuth.

After the expected size of the arbor was determined, we randomly selected the number of stems, which are the dendritic branches immediately protruding from the soma (Figure 3A), that the morphology would have based on the measured distribution. This is notable because the number of stems influences their starting orientation. For example, if the morphology had only one stem, the stem would be oriented directly upwards along the y-axis whereas multiple stems would be oriented such that they were spaced evenly around the lateral surface of a cone with its starting point at the soma and a randomly selected half angle based on the data. Gaussian noise was then added to the starting orientation of each branch so that the stems were not perfectly equidistant. Each stem was finally assigned a point process that would determine the locations of their bifurcations. Heterogeneous point processes with variable branching rates were achieved using spike thinning (Lewis and Shedler, 1979), by which a homogeneous point process with bin size delta was generated using the maximal branching rate, as described in Brown et al. (2002) and the normalized branching rate function was used to generate the probability that the bifurcation was kept at each bin given its distance from the soma.

From the initial stems, we then performed a recursive growth step. Every daughter branch from a bifurcation was assigned a new point process and was then extended by a single bin that corresponded to the next bin of its point process, given its current distance from the soma. The diameter was determined by having a set taper for the entirety of the branch. The orientation of the compartment was influenced primarily by the direction of the previous compartment, with an added random component of Gaussian noise.

### Branch Angles Based on Conditional Histograms

To establish a reference frame for branch angles, each morphology was registered to a standard coordinate plane. First, the central axis of the morphology was determined by taking the average vector of each terminal vector in a morphology. The morphology was rotated until the central axis of the morphology was aligned with the y-axis in the positive direction. At this point, the terminal field of the morphology was defined as the ellipse that fully enclosed the projection of all terminations onto the xz-plane. The major and minor axes of the morphology were defined as the major and minor axes of this ellipse. The morphology was rotated around the y-axis such that the major axis aligned with the x-axis, which completed registration.

The orientation of a branch was measured using two quantities: the bifurcation amplitude and the bifurcation azimuth. We first defined the primary and secondary daughter branches for each bifurcation. The primary daughter branch was the branch that grew in the direction most similar to the parent branch, which was determined by comparing the angles formed between each daughter branch and the parent vector. Deviation of the primary daughter from the parent branch vector was quantified as orientation noise. The bifurcation amplitude was then defined as the angle between the vectors of the primary daughter and the secondary daughter. Azimuth was defined as the clockwise angle that the secondary daughter branch needed to be rotated around the parent branch vector so that the branch would be pointed toward the central y-axis (Figure 3B). Thus, azimuth represents a branch's orientation with respect to the center of the morphology. For all branch angle problems, the branch vectors were defined by the start and end coordinates of the branch.

The bifurcation amplitude and azimuth angles were determined using 2D heatmaps (similar to **Figure 5**) that described the conditional relationships between the branch angles and parameters of interest, namely the location of the bifurcation xz-plane. Whenever a bifurcation event occurred, the x-coordinate and z-coordinate of the bifurcating compartment would be used to access the appropriate conditional distribution measured from the real data.

### Measurement of Morphometrics and Generation Procedure

The 3D reconstructions of 43 rat dentate gyrus granule cells (Rihn and Claiborne, 1990) were used as the standard for granule cell dendritic morphology. This data is publicly available on Neuromorpho.org (Ascoli et al., 2007). This dataset was selected as it contained the largest number of exemplary reconstructions of granule cells and is a highly cited authority for both qualitative and quantitative descriptions of rat granule cell dendritic morphology. Data from a single study were used because the various datasets had moderate differences in the morphometric distributions due to the differences in experimental techniques, including animal age, target selection of staining method, normalization protocol for tissue shrinkage, and handling of cut dendrites. A second dataset (Cannon et al., 1998) of 37 rat dentate gyrus granule cells was used to validate the branching rate estimate characteristics observed in the first dataset.

L-Neuron was used to generate a set of 50 granule cell dendritic morphologies for comparison. To obtain the morphometric parameters required for L-Neuron, L-Measure (Scorcioni et al., 2008) was used to extract the mean, standard deviation, and lower and upper bounds of the distributions (Supplementary Table 1). These were used as the inputs for the L-Neuron software, which generates dendritic morphologies according to the Hillman algorithm. The Hillman model involves creating a branch with an initial diameter, selecting a pathlength and taper for that branch from the appropriate distributions determined by L-Measure, and then comparing the diameter at the end of the branch to a threshold value to determine whether or not the branch should bifurcate or not. If so, two daughter branches are created with their initial diameter some ratio of the parent branch's end diameter, i.e., the daughter ratio, at branch angles randomly selected from the population distribution.

SHAPED was then used to obtain the branch rate estimate from the real data and then generate 50 granule cell dendritic morphologies using a modified Hillman algorithm. Our software was used to obtain the morphometrics relevant to SHAPED, such as the terminal point processes and conditional branch angle distributions. The rest of the algorithm follows a Hillman-like algorithm in which pathlength is selected via the randomly generated heterogenous point processes and the branch angles are assigned according to the marginal distribution corresponding to the bins of the x and z coordinates. Tapering is implemented in the same manner as in the Hillman method, but is not used as the termination criterion.

A final set of 50 granule cell dendritic morphologies were generated using a control version of SHAPED that used the same branching rate at all distances from the soma. This was referred to as the homogeneous rate method. Generated morphologies from all methods were screened to ensure the number of bifurcations fell within a biologically realistic range based on the real granule cell reconstructions. Additionally, the major and minor axes of the ellipse formed by the dendritic tips from “birds-eye-view” onto the xz-plane were measured. If the morphologies did not conform to the biologically observed range of major and minor axes, the morphologies were rescaled in the x and z directions to better distribute the dendrites at the appropriate depths in relative to the location of the soma.

## Results

**Figure 4:**
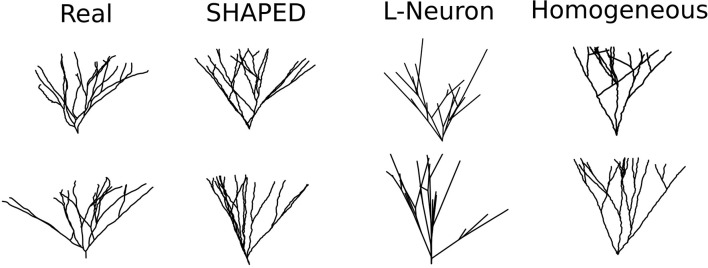
Example dendritic morphologies from various generation approaches. (1) Reconstructions of real rat dentate gyrus granule cell dendrites. (2) SHAPED morphologies. (3) L-Neuron morphologies. (4) Morphologies generated using a control version of SHAPED that applies a homogeneous branching rate at all points in the arbor.

Fifty granule cell morphologies were generated using three different methods: SHAPED, L-Neuron running an implementation of the Hillman algorithm, and a control version of SHAPED using a homogeneous branching rate. A visual comparison of representative morphologies from each dataset is seen in Figure 4.

### Reproduction of Morphometric Distributions

Table 1 presents the performance of the different generation methods presented as the root mean squared error (RMSE) between the generated morphometric distributions and the distributions of the real granule cells. The SHAPED morphologies are statistically more similar to the real granule cell morphologies, with a lower RMSE for the 15 metrics queried. In particular, the average RMSE for emergent properties was 15% lower for SHAPED than L-Neuron morphologies and 5% lower than the homogeneous rate morphologies. Basic parameter distributions were more comparable to the other methods, with L-Neuron outperforming SHAPED slightly in being able to capture the exact distributions.

**Table 1:** The Root Mean Squared Error (RMSE) between different generation algorithms and the real granule cell data set for various morphometric distributions.

**Distribution**	**SHAPED**	**L-Neuron**	**Homogeneous rate**
**RMSE for generative distributions**			
Branch pathlength	0.065	0.056	0.169
Azimuth	0.062	0.064	0.025
Bifurcation amplitude	0.047	0.099	0.052
**RMSE for emergent distributions**			
Number of bifurcations	0.079	0.599	0.086
Total dendritic length	0.288	0.2	0.708
Branch order	0.171	0.395	0.262
Depth	0.128	0.556	0.165
Width	0.188	0.144	0.181
**RMSE averages**			
Total average	0.145	0.256	0.208
Generative average	0.114	0.103	0.117
Emergent average	0.171	0.379	0.280

*While the average RMSE for generative parameters is comparable for all generation techniques, SHAPED has the least error in the distributions of emergent properties, particularly the number of bifurcations, and branch order*.

**Figure 5:**
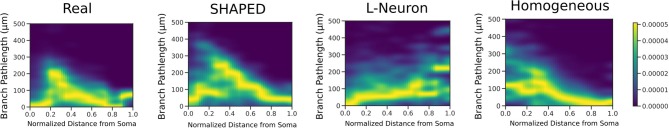
2D heatmaps displaying the conditional relationship between branch pathlength and distance from the soma. SHAPED is able to better capture the non-linear relationship observed in the real granule cell population, which is not as evident in the L-Neuron or homogeneous rate populations.

The point process filter and smoother and EM algorithm yielded the branch rate estimate in Figure 2. This was performed on two datasets (Rihn and Claiborne, 1990; Cannon et al., 1998) to confirm the branching dynamics were consistent for rat granule cells. As expected, there is a higher branching rate closer to the soma, which decays rapidly leading to a lower branch rate near the dendritic tips. The dependence of branch pathlength on the distance from the soma for the different generation techniques was visualized in detail using a smoothed heatmap (Figure 5). Each column in the histogram was normalized to generate the conditional probability mass function for each distance. The plots demonstrate that SHAPED is able to capture the heterogeneous branch rate that is present in the reconstructions while L-Neuron does not.

**Figure 6:**
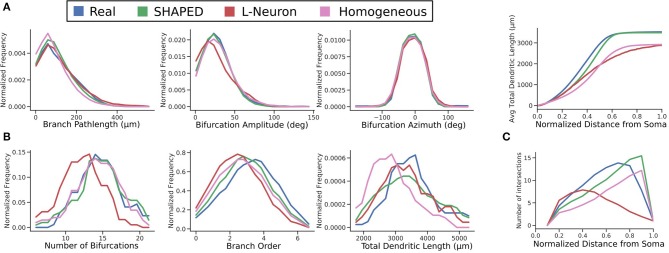
Comparison of distributions between the real (blue), SHAPED (green), L-Neuron (red), and homogeneous rate (pink) morphologies for **(A)** generative parameters explicitly used in the Hillman generation algorithm, **(B)** emergent properties observed in the finished morphologies, and **(C)** the evolution of emergent properties with increasing distance from the soma. Top: the cumulative distribution function of total dendritic length. Bottom: Sholl analysis.

The distributions for branch pathlength, bifurcation amplitude, and bifurcation azimuth are compared in Figure 6. All algorithms have similar results for the generative distributions. For the Hillman approach, this is expected because it samples directly from explicitly measured distributions, the generative parameters must result in similar distributions. On the other hand, SHAPED draws from a heterogeneous distribution, yet the homogeneous evaluation of its generative parameters result in a distribution similar to the reconstructions.

Screening plays an important role in ensuring that the number of bifurcations observed in generated morphologies are similar to the reconstructions in terms of mean and variance. One advantage of SHAPED is that the starting distribution for the number of bifurcations was appropriate such that the post-screening distribution matches closely with that of the real data, with 53% of generated neurons passing screening criteria. In the L-Neuron morphologies, the starting distribution was so far skewed that morphologies with an appropriate number of bifurcations were only generated 8% of the time. The larger proportion of SHAPED morphologies that passed the screening indicates that this method better constrains the morphologies to match the emergent properties than L-Neuron or the homogeneous rate morphologies.

A Sholl analysis (Sholl, 1953), which counts the intersections between the morphology and concentric spheres of increasing radii, was performed (Figure 6C). Both the reconstructed and SHAPED morphologies had a maximum of 14 intersections, with a fairly comparable increase in intersections with increasing distance from the soma. Most significantly, the SHAPED morphologies, on average, have slightly lower degree of early branching and a much steeper termination curve toward the boundary layer of the morphologies, due to the strict termination criterion. The L-Neuron morphologies show a much lower maximum number of intersections and a shallower termination curve, which corresponds to decreased branching rates in the middle sections of the morphologies and a large variability in dendrite termination lengths.

A direct comparison of the distributions for total dendritic length (TDL) suggests that L-Neuron generates dendritic morphologies with more realistic total dendritic lengths. The morphologies generated with SHAPED resulted in greater variance of TDL, with some morphologies having a TDL below or above the biologically observed range. However, because SHAPED captures the heterogenous branching rate, it is likely that the distribution of dendritic length was affected. Both the probability and cumulative distribution functions for total dendritic length as a function of distance from the soma were calculated (Figure 6C). The SHAPED morphologies best match the distribution of dendritic length out of all the methods. Functionally, this results in a more accurate distribution and total number of spines along generated virtual neuron models. Overall, both the generated and real data sets show a logistic increase in total dendritic length, with a steeper increase than the L-Neuron morphologies. This means that there is less branching in the L-Neuron morphologies resulting in lower average total dendritic lengths at each y-coordinate. Additionally, L-Neuron's poor termination criteria and resulting uneven branches/termination fields translate to a slowed approach to the asymptote value when compared to Claiborne and generated morphology sets.

### Reproduction of Passive Electrical Properties

**Figure 7:**
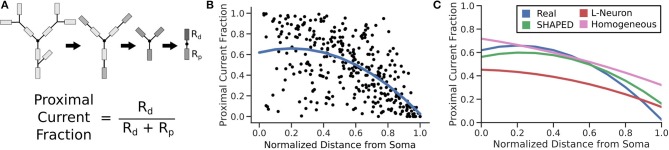
**(A)** For 100 points on each morphology, the fraction of current that would passively flow toward the soma is determined by simplifying all branches above and below the compartment into two impedances. **(B)** The resulting proximal current fractions are plotted against the normalized distance from the soma and then fit with a quadratic regression line. **(C)** The regression lines for different morphology populations are finally compared, calculating *r*^2^ to determine how much of the real dataset each fit is able to explain. (*r*^2^ values: Real = 0.359; SHAPED = 0.359; L-Neuron = 0.350; Homogeneous Rate = 0.335).

The passive electrical properties of the morphologies were assessed by calculating the equivalent resistance between a location on the morphology and the soma. The equivalent resistance between the same point and the morphology distal to that point was also computed. The proportion of the resistance that led to the soma indicated the fraction of the current that would flow toward the soma. The equivalent resistance was calculated by modeling each branch as a resistor and combining them in series and parallel. The resistance for each branch was assumed to be identical, so the calculated proportion compares the complexity of the morphology proximal to the chosen location to the complexity of the morphology distal to the chosen location. For each morphology, 100 points randomly distributed across the dendritic arbor were evaluated. The distributions of these measurements were fitted to a quadratic regression. SHAPED performed best out of the three generation techniques demonstrating that it was better able to capture the passive electrical properties of the real dendritic arbors, with an *r*^2^ = 0.359, on par with the regression line fitted directly to the Claiborne set (Figure 7).

## Discussion

**Figure 8:**
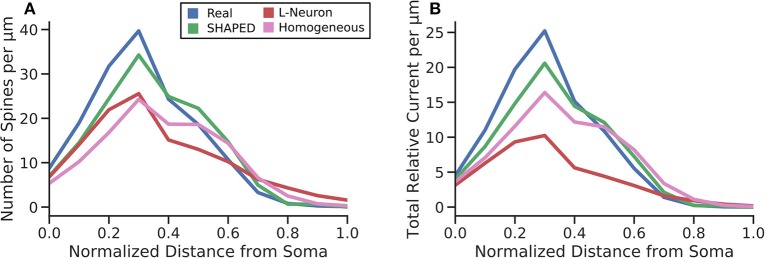
**(A)** The average number of spines each μm away from the soma. This was calculated by multiplying the probability density function of the total dendritic length per μm by spine density values for the inner, middle, and outer thirds of the dentate gyrus molecular layer obtained from literature. The normalized RMSE for the different techniques were as follows: SHAPED = 0.825, L-Neuron = 1.513, Homogeneous = 1.726. **(B)** The total relative current that would flow toward the soma under uniformly distributed synaptic inputs. The normalized RMSE for the different techniques were as follows: SHAPED = 0.884, L-Neuron = 2.613, Homogeneous = 1.582.

Though significant efforts have been made to collect and make available morphological reconstructions of neurons via databases such as NeuroMorpho.org, there are still too few reconstructions that can be used to represent the full variability that exists in the morphology for a neuron type whether it is the result of differences in the anatomical environment and/or the natural variability within a single anatomical environment. Morphology generation is a method to bootstrap the existing data and create additional morphologies. The current work introduces several improvements to current stochastic methods for generating dendritic morphologies. The most significant contribution was to model branching as a heterogeneous point process and to use the point process framework to both measure the changes in branching rate as a function of distance from the soma and to generate new branches using the estimated heterogeneous rate. The results demonstrate that a taper-based bifurcation algorithm and a homogeneous point process are insufficient to properly recreate the distribution of dendritic material in a morphology. Without a heterogeneous representation of branch rate, the amount of dendritic material per layer is underestimated. This has several functional implications. To define connectivity in neuronal network models, the convergence of one presynaptic population upon a postsynaptic neuron is estimated by understanding the dendritic layer to which presynaptic axons are located, the total dendritic length within the layer, and the spine density in the layer (Trommald and Hulleberg, 1997; Yu et al., 2019a). For a given generated morphology and a known spine density, the total dendritic length within the layer must be appropriate else the number of spines estimated for the layer will be incorrect. To demonstrate this, the number of spines per μm was determined by multiplying the dendritic length added per μm by the spine density values from literature (Crain et al., 1973; Desmond and Levy, 1985; Hama et al., 1989) for each third of the molecular layer. This was then multiplied by the proximal current fraction to yield the total relative current that would be experienced at the soma assuming uniformly distributed inputs of unit strength around the dendritic arbor (Figure 8). Our results indicate that SHAPED well-preserves the distribution of dendritic length across layers and therefore better preserves the number of connections present in each layer. Additionally, we have shown that the passive electrical properties are impacted which affect the distribution and propagation of currents throughout the morphology. Thus, both local dendritic computations and the input-output transformations performed by the neuron are better preserved by the morphologies generated by our proposed method. Notably, the morphologies generated using a homogeneous branching rate also improved the reproduction of biologically realistic distributions, but these changes were mostly due to the changes in termination criterion and screening process and therefore were most impactful in reproducing the behavior at distances far away from the soma. However, using the mean branching rate throughout the entirety of the dendritic arbor resulted in an underestimation of branching rate close to the soma, which severely impacted the homogeneous rate generation from producing morphologies with biologically accurate emergent properties. Though unexplored in this work, this may have further implications for predicting responses to extracellular electrical stimulation and local field potentials. These results indicate that heterogeneities in morphological parameters need to be considered when generating morphologies and, in particular, the heterogeneities in branch rate must not be ignored.

Although SHAPED represents a major improvement on previous algorithms, it still does not perfectly recreate the distributions measured from the reconstructions. A large portion of the differences in the distributions of generative parameters due to the SHAPED algorithm can be attributed to the overestimation of small branch pathlengths. This is likely due to the recounting of identical branch pathlengths that occur early in the terminal paths. An appropriate modification of the state observation variance, in which the variance is increased closer to the soma due to fewer unique observations, would be a possible improvement. A complete reworking of the state-space model could provide a better fit for the data and would involve the incorporation of history components (Barbieri et al., 2001; Frank et al., 2001) that would inform bifurcation probability based on previous bifurcations in the same terminal path or bifurcations occurring on neighboring terminal paths. The availability of additional morphologies would also aid the performance of the algorithm. The 43 Claiborne granule cells were a relatively low number of morphologies, but we decided to use morphologies from a single study to avoid any inconsistency in tissue preparation or tracing methodology.

The point process filter is able to measure dependencies between variables in a highly generalizable manner. Provided a sufficiently large training set, a wide variety of dendritic branching behavior can be achieved using this approach with minimal modification. The point process filter provides a more elegant means of capturing multiple different branching behaviors that occur along the length of the dendrite while avoiding the need to arbitrarily define and parameterize multiple anatomical zones. An even more sophisticated solution would likely be necessary to implement a truly generalizable algorithm to handle the branching angles of different cell types, but this could potentially be informed by the establishment of various trophic factors for self-avoidance and somatotropism as demonstrated in Memelli et al. (2013). All of this can potentially be incorporated into the state-space model framework.

The SHAPED algorithm can generate new morphologies that preserve, to a large extent, the morphological and functional properties of the reconstruction data sets from which properties are measured. Furthermore, it is able to generate morphologies that can conform to any anatomical boundary that may exist in a neural region. These features are especially important during the construction of large-scale, three-dimensional network models of neural systems, which are powerful tools for both investigating emergent network behavior and examining the mechanisms by which microcircuitry performs its computation (Bezaire and Soltesz, 2013; Markram et al., 2015; Hendrickson et al., 2016; Arkhipov et al., 2018; Yu et al., 2019a,b). Due to the importance of morphology on the input-output properties of individual neurons, it can be presumed the variability in morphology will have further consequences at the large-scale. In addition, a realistic three-dimensional morphology is important in simulating the neuronal response to stimulation modalities that vary in space, e.g., extracellular electrical stimulation and drug diffusion. Future work will be aimed at establishing to what extent biologically realistic aspects of morphologies contribute to the overall response of simulated networks.

## Data Availability Statement

Real cell morphologies were taken from public datasets made available at http://neuromorpho.org. Generated datasets are available on request.

## Author Contributions

ZC performed the study. ZC and GY wrote the manuscript. GY and TB advised the project.

### Conflict of Interest

The authors declare that the research was conducted in the absence of any commercial or financial relationships that could be construed as a potential conflict of interest.
