# Supplementary material for: Generation of Granule Cell Dendritic Morphologies by Estimating the Spatial Heterogeneity of Dendritic Branching
Source: Front Comput Neurosci. 2020 Apr 9;14:23. doi: 10.3389/fncom.2020.00023 (PMC7160759; doi:10.3389/fncom.2020.00023)
Supplement: Supplementary file 1 [file Table_1.DOCX]

**Supplementary Table 1.** Parameter values used in L-Neuron to generate dendritic morphologies. For Uniform distributions, Coefficient 1 is the lower bound and Coefficient 2 is the upper bound. For Gaussian distributions, Coefficient 1 is the mean and Coefficient 2 is the standard deviation. For Gamma distributions, Coefficient 1 is the shape parameter, Coefficient 2 is the scale parameter, and Coefficient 3 is the location parameter. Taper was implemented as an “m” distribution that utilizes multiple different distributions in specified proportions.

| Distribution | Units | Type | Coefficients | Min. Value | Max. Value |
| --- | --- | --- | --- | --- | --- |
| somadiam | μm | Scalar | 10 | - | - |
| bifamplitude | Degrees | Gamma | 3.1, 0.75, 8.15 | 0 | 100 |
| biforient | Degrees | Uniform | 0, 100 | 0 | 100 |
| Rall_power | - | Gaussian | 2.8, 0.8 | 0.44 | 4.56 |
| Ibf_Branch_pathlength | μm | Gamma | 1, 1.1, 75.1 | 1 | 500 |
| Stem_Diameter | μm | Gaussian | 3, 0.7 | 2 | 6 |
| treeazim | Degrees | Gaussian | 0, 86 | -180 | 180 |
| treeelev | Degrees | Gaussian | 0, 18.3 | -150 | 150 |
| Contraction | - | Scalar | 1 | - | - |
| Fragmentation | - | Scalar | 1 | - | - |
| PK |  | Gaussian | 1.8, 0.3 | 0.2 | 3 |
| Diam_threshold | μm | Gamma | 3.85, 7.49, 1 | 0.18 | 1.55 |
| Daughter_Ratio | - | Scalar | 1.85 | - | - |
| Taper_2 (75%) | - | Scalar | 0 | - | - |
| Taper_2 (10%) | - | Scalar | 0.2 | - | - |
| Taper_2 (5%) | - | Scalar | 0.26 | - |  |
| Taper_2 (5%) | - | Gaussian | 0.4, 0.1 | 0.2 | 0.6 |
| Taper_2 (5%) | - | Gaussian | 0.17, 0.05 | 0.1 | 0.2 |
